# Effect of endothelin‐1 on the blood pressure response to acute hypoxia and hyperoxia in healthy young men

**DOI:** 10.14814/phy2.70004

**Published:** 2024-09-01

**Authors:** Anna M. Gonsalves, Sarah E. Baker, Dain W. Jacob, Jennifer L. Harper, Camila M. Manrique‐Acevedo, Jacqueline K. Limberg

**Affiliations:** ^1^ Department of Nutrition and Exercise Physiology University of Missouri Columbia Missouri USA; ^2^ Department of Anesthesiology Mayo Clinic Rochester Minnesota USA; ^3^ Dalton Cardiovascular Research Center University of Missouri Columbia Missouri USA; ^4^ Department of Medicine University of Missouri Columbia Missouri USA; ^5^ Research Service Harry S. Truman Memorial Veterans' Hospital Columbia Missouri USA

**Keywords:** blood pressure, carotid body, chemoreflex, endothelin, hypoxia

## Abstract

Endothelin‐1 (ET‐1) and its receptors are linked to increases in sensitivity of the chemoreceptors to hypoxic stress and the development of hypertension in preclinical models. We hypothesized ET receptor antagonism would lower resting blood pressure (BP) as well as the acute BP response to chemoreflex stress. Twenty‐four men (31 ± 5 years, 26 ± 3 kg/m^2^) completed two study visits (control, bosentan). On each visit, BP was assessed under three conditions: (1) normoxia (F_i_O_2_ 0.21), (2) chemoreflex excitation via hypoxia (F_i_O_2_ 0.05–0.21), (3) chemoreflex inhibition via hyperoxia (F_i_O_2_ 1.00). Bosentan increased plasma ET‐1 (0.94 ± 0.90 to 1.27 ± 0.62 pg/mL, *p* = 0.004), supporting receptor blockade. Resting diastolic (73 ± 5 to 69 ± 7 mmHg, *p* = 0.007) and mean (93 ± 7 to 88 ± 7 mmHg, *p* = 0.005) BP were reduced following bosentan compared to control with no change in systolic BP (*p* = 0.507). The mean BP response to both acute hypoxia (−0.48 ± 0.38 to −0.25 ± 0.31 mmHg/%, *p* = 0.004) and hyperoxia (area under the curve −93 ± 108 to −27 ± 66 AU, *p* = 0.018) were attenuated following bosentan. Acute ET receptor inhibition attenuates the rise in BP during chemoreflex excitation as well as the fall in BP during chemoreflex inhibition in healthy young men. These data support a role for ET‐1 in control of resting BP, possibly through a chemoreceptor‐mediated mechanism.

## 1 INTRODUCTION

Endothelins (ETs) are a family of isopeptides that promote vasoconstriction, with endothelin‐1 (ET‐1) the prevailing ligand of the ET system (Yanagisawa et al., 1988). ET‐1 primarily targets vascular smooth muscle cell ET_A_ receptors; however, ET‐1 is produced throughout the body, including in vascular endothelial cells, neurons in the central nervous system, and Type 1 glomus cells within the carotid bodies (Prabhakar & Jacono, 2005). Based on the known vasoconstrictor effects of ET‐1, it is reasonable to expect ET‐1 to contribute significantly to resting blood pressure (BP), but findings remain unconclusive—some data show no effect of ET receptor antagonists on BP (Cirino et al., 1994; Douglas et al., 1992; Filep et al., 1994; Gardiner et al., 1991, 1994; Ihara et al., 1992), while others suggest a modest reduction (Bazil et al., 1992; Cardillo et al., 1999, 2002; Clozel et al., 1993; Haynes et al., 1996; Hoffman et al., 1994; McMahon et al., 1991; Nishikibe et al., 1993; Pollock & Opgenorth, 1993; Véniant et al., 1994; Weil et al., 2012). These conflicting outcomes may stem from compensatory mechanisms crucial for BP maintenance, potentially masking the overall effect of ET‐1. With this, an important limitation of prior work is the emphasis on the resting state, overlooking strong preclinical evidence highlighting the role of ET‐1 in the dynamic pressor response to acute stress (D'Angelo et al., 2010).

Endothelin‐1 and its receptors have been linked to increases in sensitivity of the chemoreceptors to hypoxic stress (Chen et al., 2000, 2002; Kuwaki et al., 1999; McQueen et al., 1994, 1995; Rey et al., 2006; Spyer et al., 1991), with preclinical models demonstrating attenuation of chemoreflex sensitization following ET‐1 receptor blockade (Pawar et al., 2009; Peng et al., 2013; Prabhakar & Jacono, 2005). Indeed, research supports a role for the ET system in the acute BP response to hypoxia in patients with severe untreated sleep apnea (Janssen et al., 2017). However, any effect of ET‐receptor blockade on BP could not be attributed to lower chemoreflex activation (Janssen et al., 2017), and bosentan (a non‐selective ET‐receptor antagonist) affected neither peripheral chemoreflex function nor BP in healthy young adults (Gujic et al., 2007). Unfortunately, prior work examined the acute hypoxic response only. Increased sensitivity of the chemoreceptors to hypoxia is not always associated with elevated chemoreceptor tone (Paton, Sobotka, et al., 2013)—for example, the ventilatory response to chemoreceptor excitation does not correlate with the response to chemoreceptor inhibition in adults with obstructive sleep apnea (Prasad et al., 2020). Given the chemoreflex response to excitation and inhibition are incongruent and independent, an important gap in knowledge remains.

With this information in mind, we sought to assess a role for ET‐1 in the maintenance of resting BP and determine whether chemoreflex control of BP would be altered with ET‐1 blockade. We hypothesized ET receptor antagonism with oral bosentan would lower resting BP in healthy young men as well as the acute BP response to hypoxia. We further hypothesized ET receptor antagonism would blunt the BP response to chemoreflex inhibition. Given hyperoxia can be used to demonstrate carotid body tonicity by acutely suppressing aberrant discharge (DeJours, 1963), we assessed beat‐to‐beat BP under three conditions: (1) normoxia (F_i_O_2_ 0.21), (2) chemoreflex excitation via acute, graded hypoxia (F_i_O_2_ 0.05–0.21), and (3) chemoreflex inhibition via transient hyperoxia (F_i_O_2_ 1.00).

## 2 METHODS

### 2.1 Participants

All experiments and procedures were approved by the Institutional Review Board at the Mayo Clinic (16‐004563) and University of Missouri (2007973), were in accordance with institutional guidelines, conformed to the Declaration of Helsinki, and were part of a registered clinical trial (NCT05146089). Resting BPs were published previously from a subset of participants (*n* = 12) testing unrelated hypotheses (Limberg et al., 2022), and analyses and results presented herein are unique and specific to the novel hypotheses raised.

Only young (<45 years of age) men without obesity (body mass index <30 kg/m^2^) were included in the present investigation. Participants with acute and/or chronic disease, as well as those taking any medications were excluded. Participants identified as the following: White/Non‐Hispanic (79%), White/Hispanic (13%), Asian (4%), Black (4%). Given risks associated with bosentan in women of child‐bearing age [reduced contraceptive effectiveness and Pregnancy Category X (Venitz et al., 2012)] as well as known sex‐ and sex‐hormone effects on ET‐1 and its receptors (Kuczmarski et al., 2021), this initial investigation was conducted in men only. Written informed consent was obtained from all participants at the screening visit, followed by resting BP, medical history, and fasting blood chemistries to confirm normal liver function (per clinical guidelines for the use of bosentan).

### 2.2 Instrumentation

Participants refrained from caffeine, alcohol, and strenuous exercise for 24‐h prior to testing. On the study day, participants arrived after an overnight fast and rested supine for instrumentation which included an electrocardiogram (lead II) and finger pulse oximeter. A 20‐gauge, 5‐cm catheter was placed in the brachial artery under aseptic conditions after local anesthesia for beat‐by‐beat arterial BP measurement and periodic blood sampling in 16 individuals. In eight individuals, BP was assessed non‐invasively using finger photoplethysmography (Human NIBP; ADInstruments) calibrated to upper arm sphygmomanometry and periodic blood samples were obtained via intravenous catheter. All participants had BPs within normal ranges at the screen visit. Given BP was monitored using two different methods, it is important to note beat‐to‐beat changes in non‐invasive measurements closely follow changes in intra‐arterial pressure (Petersen et al., 1995). Notably, the use of an arterial catheter has the potential for underdamping/resonance of the BP tracing, which can cause BP to read artificially high (Romagnoli et al., 2014).

Participants were instrumented with a mask connected to a non‐rebreathing valve. Breath‐by‐breath tidal volume (*n* = 16: Universal Ventilation meter; VacuMed, Ventura, CA, USA; *n* = 8: Unheated pneumotachograph with differential pressure amplifier, PA‐1, Series 1110; Hans Rudolph, Shawnee, KS, USA), respiratory rate, and inspired/expired gases (*n* = 16: GE Datex‐Ohmeda Cardiocap/5; GE Healthcare; *n* = 8: Gemini 14‐10000 Respiratory Monitor, CWE, Inc.) were monitored continuously and ventilation was expressed in ambient temperature, pressure saturated.

**FIGURE 1:**
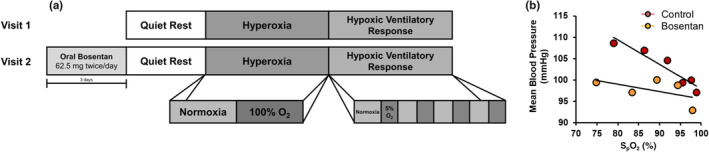
Study timeline and design. Participants completed two study visits (control, bosentan) during which they were assessed under quiet rest (normoxia, F_i_O_2_ 0.21), chemoreflex inhibition via transient hyperoxia (F_i_O_2_ 1.00), and chemoreflex excitation via acute, graded hypoxia (F_i_O_2_ 0.05–0.21) (a). Data from the transient hypoxia trial are presented as the slope of the regression line obtained from baseline and four hypoxic challenges (b).

Following a 5‐min quiet resting period and a 3‐min baseline, chemoreflex inhibition was elicited by transient hyperoxia as a modified DeJour's test, administered using a 100% oxygen gas cylinder over 2 min (Figure 1a). A 50‐L meteorological balloon served as a volume reservoir. With this, we anticipated a 1–4 mmHg reduction in mean BP based on prior studies showing similar reductions (Paula‐Ribeiro et al., 2019; Sinski et al., 2014; Stickland et al., 2011). Due to equipment issues, paired data are available from *n* = 21 during acute hyperoxia.

Following a minimum 4‐min washout period and 2‐min normoxic baseline, chemoreflex excitation was produced by acute, graded hypoxia achieved by alternating between a hypercapnic hypoxic (5% oxygen, 3% carbon dioxide) gas cylinder and room air (21% oxygen). Tests took approximately 15 min to complete. Normoxic measures were averaged from the 10‐breath period prior to beginning the series of hypoxic challenges, at which time variable levels of hypoxemia were achieved using two to six breaths of hypoxic air followed by room air using methods published previously (Chua & Coats, 1995; Limberg et al., 2016; Paula‐Ribeiro et al., 2019, 2021). This was repeated four times, with the goal of achieving oxygen saturation values ranging from 70% to 99% (Limberg et al., 2016).

### 2.3 ET receptor inhibition

Participants completed two study visits (control, bosentan), separated by at least 1 week (Range 7–62 days; Average = 31 ± 15 days) with visits completed at the same time of day. Control visits were completed first. For 3 days prior to the second visit, participants consumed 62.5 mg of bosentan (Tracleer; Actelion Pharmaceuticals) by mouth twice daily at home for a total dose of 125 mg per day. A final dose (62.5 mg) was ingested the morning of the study and data collection began 3 h after intake of the final dose. This dosing was based on prior work showing peak plasma concentrations of bosentan are achieved after 3–5 h, the half‐life is 5 h, and steady‐state concentrations are achieved by 3 days (Venitz et al., 2012). A comparable dose of bosentan has been shown previously to effectively achieve ET‐receptor blockade (Hiramoto et al., 2007) and has thusly been applied by other groups (Gujic et al., 2007). Compliance with bosentan was confirmed on the study day via self‐report. Additionally, plasma ET‐1 was assessed using commercially available kits according to manufacturer's protocols at the Mayo Clinic (*n* = 16: QET00B; R&D Systems, Inc., Minneapolis, MN, USA) and University of Missouri (*n* = 7: DET100; R&D Systems, Inc.).

### 2.4 Data analysis

Data were recorded at 1000 Hz using a computer data acquisition system (PowerLab; ADInstruments) and stored for off‐line analysis. Resting data were analyzed over an approximate 5‐min baseline period. During transient hypoxia, data were analyzed breath‐by‐breath, as published previously (Limberg et al., 2016). Data were inspected in real time for artifacts (i.e., coughing, movement, etc.) and any artifacts were excluded from analysis. The nadir oxygen saturation within 1 min following each hypoxic challenge was determined. A minimum change in saturation (>1%) depicting a measurable change from baseline was required. The hypoxic ventilatory response (L/min/%) was calculated as the slope of the regression line obtained from baseline and four hypoxic challenges (five data points) for minute ventilation (largest three consecutive breaths within 1 min following each hypoxic challenge) versus oxygen saturation (S_p_O_2_). Similarly, the peak heart rate (beats/min/%) and mean BP (mmHg/%) responses were determined following each hypoxic challenge and plotted against the nadir oxygen saturation (Figure 1b). This approach has been shown previously to be repeatable across time (Chua & Coats, 1995; Limberg et al., 2016; Niewinski et al., 2013, 2014). Chemoreflex inhibition was elicited by transient hyperoxia, and data were analyzed as 15‐s averages during the first 1‐min of air administration. To account for any potential impact of changes in resting variables, both an absolute (∆ = 15 sec – Baseline) and relative (% = ∆ ÷ Baseline × 100) change from baseline were calculated and area under the curve (AUC) responses were assessed.

Statistical analysis was completed using SigmaPlot 14.0 (Systat Software, Inc.) and *p* < 0.05 was considered statistically significant. The effect of visit (control, bosentan) on main outcome variables was assessed using a paired *t*‐test, unless not normally distributed (Shapiro–Wilk), when data were compared using Wilcoxon Signed Rank Test. The main effects of visit (control, bosentan) and time, and the interaction of visit and time on the acute response to hyperoxia, was assessed using a two‐way repeated measures analysis of variance (two factor repetition) with normality testing (Shapiro–Wilk). Pairwise comparisons were done with the Bonferroni *t*‐test. Data are reported as mean ± standard deviation unless otherwise noted.

## 3 RESULTS

**FIGURE 2:**
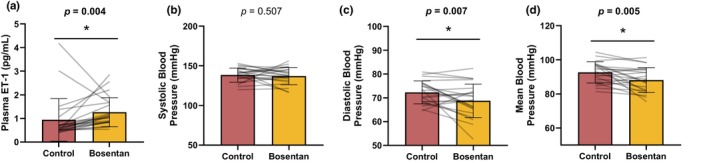
Impact of bosentan on endotheline‐1 (a) and resting systolic (b), diastolic (c), and mean (d) blood pressure. Data are reported as mean ± standard deviation from *n* = 24 men. Data are compared using paired *t*‐test. *p < 0.05 Control versus bosentan.

Results are reported from 24 young (31 ± 5 years), normotensive (Screen visit: 117 ± 9/70 ± 8 mmHg) men without obesity (26 ± 3 kg/m^2^). Oral bosentan resulted in an increase in plasma ET‐1 (Figure 2a, *p* = 0.004), supporting effectiveness of receptor blockade. As shown previously from a subset (*n* = 12) of these participants (Limberg et al., 2022), and confirmed in this larger cohort, resting diastolic (*p* = 0.007) and mean (*p* = 0.005) BP were reduced following 3 days of bosentan treatment compared to control with no change in systolic BP (*p* = 0.507) (Figure 2b–d). No changes in resting heart rate (56 ± 8 to 57 ± 7 beats/min, *p* = 0.108) or minute ventilation (6.6 ± 2.0 to 7.5 ± 3.0 L/min, *p* = 0.131) were observed following treatment with bosentan.

**FIGURE 3:**
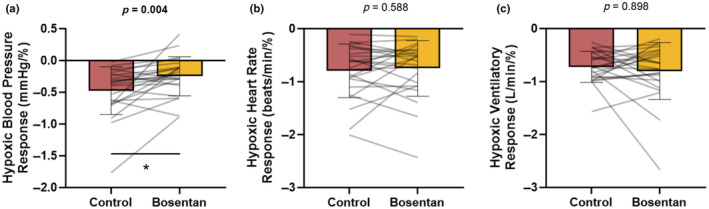
Impact of bosentan on the blood pressure (a), heart rate (b), and ventilatory (C) response to transient hypoxia. Data are reported as mean ± standard deviation from *n* = 24 men. Data are compared using paired *t*‐test, unless not normally distributed (Shapiro–Wilk), when data are compared using Wilcoxon Signed Rank Test (a, c). *p < 0.05 Control versus bosentan.

**FIGURE 4:**
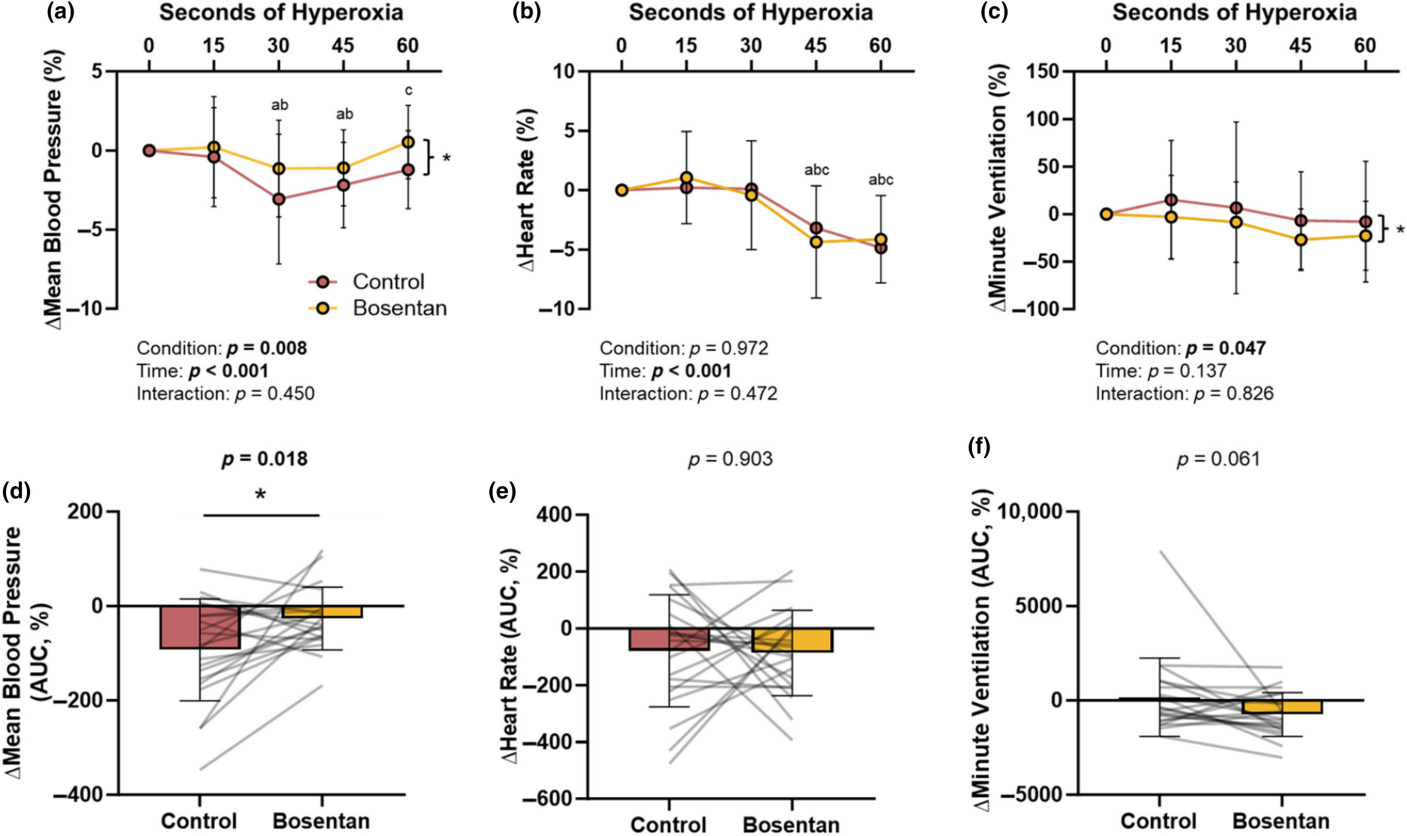
Impact of bosentan on the relative (%) and relative area under the curve (AUC) responses to acute hyperoxia. Data are reported as mean ± standard deviation from *n* = 21 men. Relative data (a–c) are compared using two‐way repeated measures analysis of variance (two factor repetition). Relative AUC data (d–f) are compared using paired *t*‐test unless noted (Minute Ventilation: Wilcoxon Signed Rank test). ^a^ *p* <0.05 versus T0, ^b^ *p* <0.05 versus T15, ^c^ *p* <0.05 versus T30. *p < 0.05 Main effect of condition (Control vs. Bosentan).

Consistent with our hypothesis, the mean BP response to acute hypoxia was attenuated following bosentan (−0.48 ± 0.38 to −0.25 ± 0.31 mmHg/%, *p* = 0.004; Figure 3a). The fall in mean BP during acute hyperoxia was also attenuated following ET‐1 receptor blockade (main effect of bosentan, *p* = 0.007; main effect of time, *p* < 0.001; interaction of bosentan and time, *p* = 0.420; data not shown). Conclusions during hyperoxia were maintained when changes in BP were assessed as both AUC (*p* = 0.014; data not shown) and as a relative (%) change from the new, lower baseline (main effect of bosentan, *p* = 0.008; main effect of time, *p* < 0.001; interaction of bosentan and time, *p* = 0.450; Figure 4a; % AUC *p* = 0.018; Figure 4d).

There was no effect of bosentan on the heart rate response to acute hypoxia (−0.80 ± 0.51 to −0.75 ± 0.52 beats/min/%, *p* = 0.588; Figure 3b) nor hyperoxia (main effect of bosentan, *p* = 0.817; main effect of time, *p* < 0.001; interaction of condition and time, *p* = 0.396; data not shown). Conclusions during hyperoxia were maintained when changes in heart rate were assessed as both AUC (*p* = 0.394; data not shown) and as a relative (%) change from the baseline (main effect of bosentan, *p* = 0.972; main effect of time, *p* < 0.001; interaction of bosentan and time, *p* = 0.472, Figure 4b; % AUC *p* = 0.903; Figure 4e).

There was no effect of condition on the ventilatory response to acute hypoxia (−0.72 ± 0.29 to −0.80 ± 0.54 L/min/%, *p* = 0.898; Figure 3c). Hyperoxia resulted in a transient reduction in minute ventilation which was augmented following bosentan compared to control (main effect of bosentan, *p* = 0.014; main effect of time, *p* = 0.084; interaction of condition and time, *p* = 0.592; data not shown). When controlling for the effect of bosentan on resting minute ventilation (% change from baseline), conclusions were maintained (main effect of bosentan, *p* = 0.047; main effect of time, *p* = 0.137; interaction of bosentan and time, *p* = 0.826; Figure 4c), although significance was lost when changes in ventilation were assessed as AUC (∆AUC, *p* = 0.069, data not shown; %AUC, *p* = 0.061; Figure 4f).

## 4 DISCUSSION

Herein we demonstrate ET‐receptor blockade lowers resting mean arterial BP in young men. These data reiterate previous results from our group from a smaller cohort (Limberg et al., 2022) and confirm results from others (Haynes et al., 1996). We expand upon these findings and show the effect of ET receptor inhibition with bosentan further attenuates both: (1) the rise in BP during chemoreflex excitation via acute, graded hypoxia, as well as (2) the fall in BP during chemoreflex inhibition (transient hyperoxia). Together these new findings support a role for ET‐1 in control of BP in healthy young men, possibly through a chemoreceptor‐mediated mechanism.

### 4.1 ET‐1 and BP regulation

Bosentan acts as a nonspecific antagonist for ET‐receptors and as such its actions are mediated by both ET_A_ and ET_B_ receptors. Elevations in plasma ET‐1 following 3 days of oral bosentan were observed, supporting effective receptor blockade and participant compliance. Our data demonstrate 3 days of oral bosentan is sufficient to lower resting diastolic and mean BP in healthy young adult men, despite no change in systolic BP. These data confirm prior work from our group (Limberg et al., 2022), now in a larger cohort. Gujic et al. (2007) similarly demonstrated no effect of bosentan on systolic BP in a comparable group of younger men (range 20–29 years); unfortunately, diastolic BP and mean BP were not reported. Based on the lack of an effect of bosentan on heart rate nor systolic BP, we speculate any effect of bosentan on BP can be attributed to its role in regulating vascular tone, as opposed to cardiac output.

### 4.2 Chemoreflex sensitivity and basal support of BP

Vascular tone is regulated on a beat‐by‐beat basis by the sympathetic nervous system, which is under tonic reflex control (e.g., baroreflex, chemoreflex). In addition to direct effects on the vascular smooth muscle, ET‐1 and its receptors have been linked to increased hypoxic sensitivity of the chemoreceptors (Chen et al., 2000, 2002; Kuwaki et al., 1999; McQueen et al., 1994, 1995; Rey et al., 2006; Spyer et al., 1991), which is attenuated following ET‐1 receptor blockade (Pawar et al., 2009; Peng et al., 2013; Prabhakar & Jacono, 2005). With this information in mind, we sought to determine whether chemoreflex control of BP would be altered with ET‐1 blockade in heathy humans. To address this question, we applied a transient hypoxic stimulus and continuously monitored heart rate, BP, and ventilation. Our data show acute ET‐1 receptor antagonism in healthy young men attenuates the mean BP response to hypoxia independent of changes in heart rate or minute ventilation. Notably, Gujic et al. (2007) found no effect of oral bosentan on the systolic BP response to 5‐min of continuous hypoxia in younger men. Discrepancies between results may be due to lack of continuous BP monitoring which limits the ability to examine reflex changes independent of potential compensatory responses (e.g., baroreflex activation). Furthermore, the response to hypoxia is biphasic and continued exposure to low oxygen can result in a decrease in ventilation [i.e., hypoxic ventilatory decline (Easton et al., 1986)], thus the approach by Gujic et al may have masked the effect of the peripheral chemoreceptors. With this, a strength of the present investigation was the examination of the acute, peak response to transient hypoxia which has been shown by our group and others to be reproducible (Chua & Coats, 1995; Limberg et al., 2016; Niewinski et al., 2013, 2014).

In alignment with our findings, data from older men with severe sleep apnea (average age: 50 ± 9 years) support a role for the ET system in the systolic BP response to hypoxia (Janssen et al., 2017). Interestingly, although the BP response to hypoxia was attenuated following bosentan treatment, the heart rate and ventilatory responses were preserved (Janssen et al., 2017)—leading the authors to conclude the effect of bosentan on BP was not due to lower chemoreflex activation. We similarly did not observe an effect of ET‐1 receptor inhibition on the ventilatory or heart rate response to hypoxia. Rather than concluding from these data that the chemoreflex does not contribute to BP, it has been postulated more recently that sub‐populations of glomus cells exist which channel separately into reflex pathways controlling distinct target organs (Paton, Ratcliffe, et al., 2013)—thus chemoreflex‐mediated changes in BP may occur independently of changes in ventilation.

Notably, carotid body tonicity can occur in absence of increased chemosensitivity (Paton, Sobotka, et al., 2013). To address this possibility, we comprehensively assessed a role for ET‐1 on tonic chemoreflex support of BP using acute hyperoxia—which has not been explored previously. Applying acute hyperoxia in the form of a modified DeJours test (Dejours, 1963; Stickland et al., 2011), we show ET receptor blockade with bosentan attenuates the fall in BP during chemoreflex inhibition. The inclusion of a hyperoxia trial to elicit chemoreflex inhibition allows for a thorough understanding of the scope of chemoreflex support of BP. Combined findings support a role for ET‐1 in the maintenance of resting BP in the individuals studied which occurs, at least in part, through a chemoreceptor‐mediated mechanism.

### 4.3 Experimental considerations

Neither investigators nor study participants were blinded to study conditions as study visits were not randomly assigned, a decision required by the Bosentan Risk Evaluation and Mitigation Strategy (REMS) program. Thus, it is possible the lack of condition blindness could have affected findings in unexpected ways. Similarly, trial order (hyperoxia, hypoxia) were not randomly assigned and remained consistent between participants and across visits. Importantly, prior work has shown a 4‐min washout period is sufficient to prevent carryover to subsequent experimental trials (Stickland et al., 2011). Only men were studied due to the teratogenic nature of the study drug, and the generalization of results to women is not likely (Sebzda et al., 2018). Lastly, although our data support effective receptor blockade (Hiramoto et al., 2007), the dosage of bosentan administered (125 mg/day) was conservative, and other groups have recently applied higher dosages (Derella et al., 2022). It is also important to acknowledge ET‐1 potentiates vasoconstriction to norepinephrine in the human microcirculation, such that inhibition of ET_A_ receptors may attenuate sympathetic‐mediated vasoconstriction (Gössl et al., 2004; Wenzel et al., 2001). With this, it is possible ET‐1 receptor blockade with oral bosentan exhibits an effect on chemoreflex control of BP which is independent of direct action on the peripheral chemoreceptors; future work examining the pressor response to another sympathoexcitatory maneuver could address this possibility.

## 5 CONCLUSION

Acute ET‐receptor blockade with oral bosentan reduces resting BP in young men. Consistent with our hypothesis, we show ET‐receptor inhibition attenuates the rise in BP during chemoreflex excitation via acute, graded hypoxia as well as the fall in BP during chemoreflex inhibition (transient hyperoxia). The present study fills a key gap in knowledge and supports a role for ET‐1 in control of resting BP in young men, possibly through a chemoreceptor‐mediated mechanism. Future work will be necessary to determine whether these results translate to other study populations (e.g., women).

## FUNDING INFORMATION

The study was funded by NIH HL130339 (JKL), NIH U54 AG044170 (SEB), Mayo Clinic Center for Biomedical Discovery (JKL).

## CONFLICT OF INTEREST STATEMENT

The authors declare no conflicts of interest.

## Data Availability

The data that support the findings of this study are available from the corresponding author upon reasonable request.
